# The Half-Yearly Abstract of the Medical Sciences

**Published:** 1852-04

**Authors:** 


### BIBLIOGRAPHICAL.

*The Half-Yearly Abstract of the Medical Sciences.* Edited by G. W. H. Rankin, M. D. Philadelphia: Lindsay & Blakiston. No. 14, July to December, 1851.

Of the making of books, there is no end, and of the reading of them all, even of the good ones, there is no possibility. To the medical practitioner especially, the effort to keep up with the press is utterly hopeless. Hence, such publications as the excellent one before us are most necessary and benevolent devices. We recommend Rankin's Abstract to all who do not take it. It will furnish all the best papers from all the best journals. Each number contains 296 pages, and is furnished to distant subscribers, free of postage, at the very low price of two dollars per annum, and it is worth *double the amount.*

